# (Acetyl­acetonato-κ^2^ *O*,*O*′)bis­[2-(5-methyl-3-phenyl­pyrazin-2-yl-κ*N* ^1^)phen­yl-κ*C* ^1^]iridium(III)

**DOI:** 10.1107/S1600536812006022

**Published:** 2012-02-17

**Authors:** Guo-Ping Ge, Chun-Yan Li, Cheng-Hao Gu, Mao-He Li, Xiao-Nan Xu

**Affiliations:** a State Key Laboratory Base of Novel Functional Materials and Preparation Science, Institute of Solid Materials Chemistry, Faculty of Materials Science and Chemical Engineering, Ningbo University, Ningbo 315211, People’s Republic of China

## Abstract

In the title complex, [Ir(C_17_H_13_N_2_)_2_(C_5_H_7_O_2_)], the Ir^III^ atom is hexa­coordinated in a distorted octa­hedral geometry by two *C*,*N*-bidentate 2-(5-methyl-3-phenyl­pyrazin-2-yl)phenyl (mdpp) ligands and one O,O-bidentate acetyl­acetonate ligand. The dihedral angles between the phenyl rings and the pyrazine ring are 9.56 (14) and 58.99 (14)° for one mdpp ligand and 9.34 (14) and 79.94 (15)° for the other.

## Related literature

For background to organic light-emitting diodes based on phospho­rescent complexes, see: Baldo *et al.* (1998 ▶, 2000 ▶); Hwang *et al.* (2005 ▶); Liu *et al.* (2008 ▶); Tsuboyama *et al.* (2003 ▶). For the synthesis, see: Zhang *et al.* (2003 ▶, 2005 ▶).
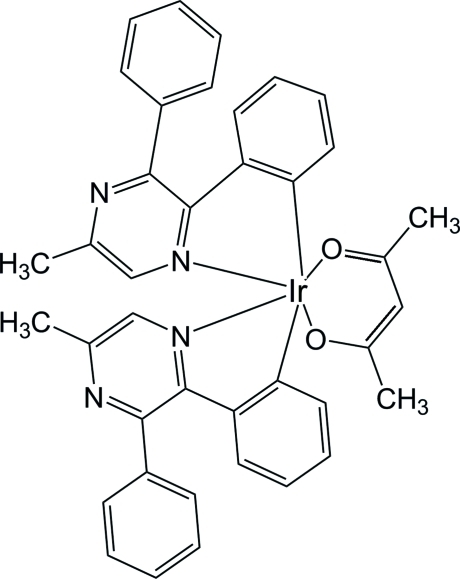


## Experimental

### 

#### Crystal data

[Ir(C_17_H_13_N_2_)_2_(C_5_H_7_O_2_)]
*M* *_r_* = 781.91
Monoclinic, 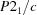

*a* = 11.078 (2) Å
*b* = 26.021 (5) Å
*c* = 12.155 (2) Å
β = 106.09 (3)°
*V* = 3366.6 (12) Å^3^
*Z* = 4
Mo *K*α radiation
μ = 4.01 mm^−1^
*T* = 295 K
0.43 × 0.30 × 0.12 mm

#### Data collection

Rigaku R-AXIS RAPID diffractometer
Absorption correction: multi-scan (*ABSCOR*; Higashi, 1995 ▶) *T*_min_ = 0.240, *T*_max_ = 0.606
25384 measured reflections
5991 independent reflections
4919 reflections with *I* > 2σ(*I*)
*R*_int_ = 0.044

#### Refinement

*R*[*F* ^2^ > 2σ(*F* ^2^)] = 0.031
*wR*(*F* ^2^) = 0.062
*S* = 1.05
5991 reflections
415 parameters
H-atom parameters constrained
Δρ_max_ = 1.12 e Å^−3^
Δρ_min_ = −0.38 e Å^−3^

### 

Data collection: *RAPID-AUTO* (Rigaku, 1998 ▶); cell refinement: *RAPID-AUTO*; data reduction: *CrystalStructure* (Rigaku/MSC, 2002 ▶); program(s) used to solve structure: *SHELXS97* (Sheldrick, 2008 ▶); program(s) used to refine structure: *SHELXL97* (Sheldrick, 2008 ▶); molecular graphics: *XP* in *SHELXTL* (Sheldrick, 2008 ▶); software used to prepare material for publication: *SHELXTL*.

## Supplementary Material

Crystal structure: contains datablock(s) global, I. DOI: 10.1107/S1600536812006022/hy2507sup1.cif

Structure factors: contains datablock(s) I. DOI: 10.1107/S1600536812006022/hy2507Isup2.hkl

Additional supplementary materials:  crystallographic information; 3D view; checkCIF report

## Figures and Tables

**Table 1:** Selected bond lengths (Å)

Ir1—N1	2.035 (3)
Ir1—N3	2.035 (3)
Ir1—O1	2.162 (3)
Ir1—O2	2.173 (3)
Ir1—C22	2.006 (4)
Ir1—C39	1.999 (4)

**Fig. 1.**
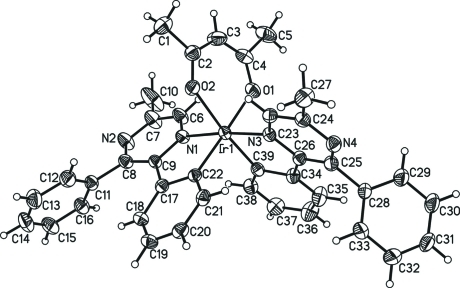
The molecular structure of the title complex, showing displacement ellipsoids at the 30% probability level.

## supplementary crystallographic information

### Comment

In recent decades, organic light-emitting diodes (OLEDs) based on phosphorescent complexes have attracted increasing attention due to their potential applications in full-color flat panel displays (Baldo *et al.*, 1998, 2000). Among these phosphorescent complexes, iridium cyclometalates often exhibit favorable photoproperties for OLEDs including short phosphorescent lifetimes, high quantum efficiencies and good stability (Liu *et al.*, 2008; Hwang *et al.*, 2005; Tsuboyama *et al.*, 2003). Zhang *et al.* (2005) demonstrated a high efficiency yellow OLED using [Ir(mdpp)_2_(acac)] [mdpp = 2-(5-methyl-3-phenylpyrazine-2-yl)phenyl, Hacac = acetylacetone] as the dopant. In this work, we synthesized the title complex, [Ir(mdpp)_2_(acac)], and investigated its crystal structure.

The molecular structure of the title complex is shown in Fig. 1. The Ir^III^ ion has an approximately octahedral coordination geometry and is hexacoordinated by two C atoms and two N atoms from two C,N-bidentate mdpp ligands, which exhibit *cis*-C,C and *trans*-N,N chelate dispositions, and two O atoms from one O,O-bidentate acac ligand. The bond lengths of Ir—N,O,C are listed in Table 1. Due to steric interactions, the phenyl groups are not coplanar with the pyrazine group. The dihedral angles between the phenyl rings and pyrazine ring are 58.99 (14)° (between the N1,N2,C6–C9 ring and C11–C16 ring), 9.56 (14)° (between the N1,N2,C6–C9 ring and C17–C22 ring), 79.94 (15)° (between the N3,N4,C23–C26 ring and C28–C33 ring) and 9.34 (14)° (between the N3,N4,C23–C26 ring and C34–C39 ring).

### Experimental

The title complex was obtained according to the procedure previously reported (Zhang *et al.*, 2003, 2005). Orange red crystals of the title complex suitable for X-ray structure analysis were grown from a mixture of 3 ml dichloromethane and 12 ml ethanol.

### Refinement

H atoms attached to C atoms were positioned geometrically and treated as riding, with C—H = 0.93 (aromatic) and 0.96 (methyl) Å and with *U*_iso_(H) = 1.2(1.5 for methyl)*U*_eq_(C). The highest residual electron density was found at 0.88 Å from Ir1 atom and the deepest hole at 1.51 Å from O1 atom.

### Crystal data

**Table:**

[Ir(C_17_H_13_N_2_)_2_(C_5_H_7_O_2_)]	*F*(000) = 1552.0
*M**_r_* = 781.91	*D*_x_ = 1.543 Mg m^−^^3^
Monoclinic, *P*2_1_/*c*	Mo *K*α radiation, λ = 0.71073 Å
Hall symbol: -P 2ybc	Cell parameters from 6987 reflections
*a* = 11.078 (2) Å	θ = 1.0–25.1°
*b* = 26.021 (5) Å	µ = 4.01 mm^−^^1^
*c* = 12.155 (2) Å	*T* = 295 K
β = 106.09 (3)°	Flaky, orange
*V* = 3366.6 (12) Å^3^	0.43 × 0.30 × 0.12 mm
*Z* = 4	

### Data collection

**Table:**

Rigaku R-AXIS RAPID diffractometer	5991 independent reflections
Radiation source: rotation anode	4919 reflections with *I* > 2σ(*I*)
Graphite monochromator	*R*_int_ = 0.044
ω scans	θ_max_ = 25.1°, θ_min_ = 3.0°
Absorption correction: multi-scan (*ABSCOR*; Higashi, 1995)	*h* = −13→12
*T*_min_ = 0.240, *T*_max_ = 0.606	*k* = −31→31
25384 measured reflections	*l* = −14→14

### Refinement

**Table:**

Refinement on *F*^2^	Primary atom site location: structure-invariant direct methods
Least-squares matrix: full	Secondary atom site location: difference Fourier map
*R*[*F*^2^ > 2σ(*F*^2^)] = 0.031	Hydrogen site location: inferred from neighbouring sites
*wR*(*F*^2^) = 0.062	H-atom parameters constrained
*S* = 1.05	*w* = 1/[σ^2^(*F*_o_^2^) + (0.0275*P*)^2^ + 3.1586*P*] where *P* = (*F*_o_^2^ + 2*F*_c_^2^)/3
5991 reflections	(Δ/σ)_max_ = 0.001
415 parameters	Δρ_max_ = 1.12 e Å^−^^3^
0 restraints	Δρ_min_ = −0.38 e Å^−^^3^

### Special details

**Table:**

Geometry. All e.s.d.'s (except the e.s.d. in the dihedral angle between two l.s. planes) are estimated using the full covariance matrix. The cell e.s.d.'s are taken into account individually in the estimation of e.s.d.'s in distances, angles and torsion angles; correlations between e.s.d.'s in cell parameters are only used when they are defined by crystal symmetry. An approximate (isotropic) treatment of cell e.s.d.'s is used for estimating e.s.d.'s involving l.s. planes.
Refinement. Refinement of *F*^2^ against ALL reflections. The weighted *R*-factor *wR* and goodness of fit *S* are based on *F*^2^, conventional *R*-factors *R* are based on *F*, with *F* set to zero for negative *F*^2^. The threshold expression of *F*^2^ > σ(*F*^2^) is used only for calculating *R*-factors(gt) *etc*. and is not relevant to the choice of reflections for refinement. *R*-factors based on *F*^2^ are statistically about twice as large as those based on *F*, and *R*-factors based on ALL data will be even larger.

### Fractional atomic coordinates and isotropic or equivalent isotropic displacement parameters (Å^2^)

**Table:**

	*x*	*y*	*z*	*U*_iso_*/*U*_eq_	
Ir1	0.208032 (15)	0.139098 (6)	0.782061 (14)	0.03722 (6)	
C1	0.4386 (5)	0.1537 (2)	1.1455 (4)	0.0693 (15)	
H22A	0.5054	0.1691	1.1209	0.104*	
H22B	0.4668	0.1217	1.1833	0.104*	
H22C	0.4140	0.1764	1.1977	0.104*	
C2	0.3278 (4)	0.14403 (17)	1.0431 (4)	0.0525 (11)	
C3	0.2208 (5)	0.1215 (2)	1.0628 (4)	0.0698 (15)	
H20A	0.2264	0.1140	1.1389	0.084*	
C4	0.1087 (5)	0.10886 (19)	0.9852 (5)	0.0580 (13)	
C5	0.0044 (5)	0.0863 (2)	1.0299 (5)	0.0805 (17)	
H19A	−0.0679	0.0794	0.9668	0.121*	
H19B	−0.0177	0.1104	1.0810	0.121*	
H19C	0.0330	0.0549	1.0702	0.121*	
C6	0.2705 (5)	0.02880 (17)	0.8455 (4)	0.0589 (13)	
H2A	0.2061	0.0309	0.8808	0.071*	
C7	0.3347 (5)	−0.01666 (18)	0.8502 (5)	0.0722 (16)	
C8	0.4656 (4)	0.02194 (16)	0.7558 (4)	0.0475 (11)	
C9	0.3949 (4)	0.06790 (15)	0.7408 (3)	0.0393 (9)	
C10	0.3003 (7)	−0.0642 (2)	0.9060 (7)	0.123 (3)	
H39A	0.3558	−0.0918	0.9003	0.185*	
H39B	0.2153	−0.0738	0.8680	0.185*	
H39C	0.3079	−0.0572	0.9852	0.185*	
C11	0.5840 (4)	0.01549 (16)	0.7211 (4)	0.0468 (11)	
C12	0.6876 (4)	0.0471 (2)	0.7647 (4)	0.0627 (14)	
H17A	0.6831	0.0734	0.8151	0.075*	
C13	0.7983 (5)	0.0394 (2)	0.7333 (5)	0.0747 (16)	
H13A	0.8672	0.0607	0.7621	0.090*	
C14	0.8042 (5)	0.0002 (2)	0.6596 (6)	0.0769 (17)	
H14A	0.8784	−0.0056	0.6399	0.092*	
C15	0.7021 (5)	−0.0308 (2)	0.6146 (5)	0.0705 (15)	
H15A	0.7062	−0.0564	0.5624	0.085*	
C16	0.5932 (5)	−0.02378 (18)	0.6470 (4)	0.0572 (12)	
H16A	0.5254	−0.0457	0.6187	0.069*	
C17	0.4081 (4)	0.11465 (16)	0.6771 (3)	0.0394 (9)	
C18	0.4850 (4)	0.11940 (17)	0.6030 (4)	0.0443 (10)	
H8A	0.5310	0.0914	0.5896	0.053*	
C19	0.4920 (4)	0.16631 (17)	0.5498 (4)	0.0496 (11)	
H4A	0.5432	0.1697	0.5011	0.059*	
C20	0.4227 (4)	0.20772 (17)	0.5693 (4)	0.0488 (11)	
H10A	0.4304	0.2393	0.5363	0.059*	
C21	0.3419 (4)	0.20268 (16)	0.6377 (4)	0.0464 (10)	
H11A	0.2948	0.2309	0.6479	0.056*	
C22	0.3292 (4)	0.15624 (15)	0.6917 (3)	0.0377 (9)	
C23	0.1626 (4)	0.24994 (16)	0.8225 (4)	0.0428 (10)	
H28A	0.2327	0.2468	0.8854	0.051*	
C24	0.1039 (4)	0.29742 (16)	0.7977 (4)	0.0450 (10)	
C25	−0.0375 (4)	0.26227 (16)	0.6396 (4)	0.0437 (10)	
C26	0.0176 (4)	0.21332 (15)	0.6644 (4)	0.0403 (9)	
C27	0.1525 (5)	0.34458 (17)	0.8674 (4)	0.0650 (14)	
H34A	0.0994	0.3733	0.8369	0.098*	
H34B	0.2366	0.3516	0.8643	0.098*	
H34C	0.1525	0.3389	0.9454	0.098*	
C28	−0.1423 (4)	0.27482 (16)	0.5357 (4)	0.0444 (10)	
C29	−0.2639 (4)	0.2821 (2)	0.5423 (5)	0.0689 (15)	
H33A	−0.2825	0.2773	0.6117	0.083*	
C30	−0.3581 (5)	0.2964 (2)	0.4461 (5)	0.0759 (17)	
H35A	−0.4398	0.3009	0.4508	0.091*	
C31	−0.3309 (5)	0.3038 (2)	0.3450 (5)	0.0669 (14)	
H36A	−0.3950	0.3122	0.2800	0.080*	
C32	−0.2114 (5)	0.2992 (2)	0.3375 (5)	0.0765 (16)	
H37A	−0.1928	0.3061	0.2690	0.092*	
C33	−0.1174 (5)	0.2840 (2)	0.4334 (4)	0.0701 (15)	
H38A	−0.0358	0.2799	0.4279	0.084*	
C34	−0.0151 (4)	0.16473 (16)	0.5999 (4)	0.0448 (10)	
C35	−0.1229 (5)	0.15696 (19)	0.5085 (5)	0.0658 (14)	
H25A	−0.1818	0.1831	0.4863	0.079*	
C36	−0.1418 (5)	0.1103 (2)	0.4511 (5)	0.0747 (16)	
H30A	−0.2135	0.1049	0.3909	0.090*	
C37	−0.0527 (5)	0.07180 (18)	0.4845 (4)	0.0626 (13)	
H24A	−0.0638	0.0409	0.4445	0.075*	
C38	0.0516 (4)	0.07848 (16)	0.5754 (4)	0.0493 (11)	
H23A	0.1096	0.0519	0.5960	0.059*	
C39	0.0730 (4)	0.12449 (15)	0.6381 (4)	0.0411 (10)	
N1	0.2983 (3)	0.07045 (12)	0.7911 (3)	0.0414 (8)	
N2	0.4340 (4)	−0.01988 (14)	0.8071 (4)	0.0649 (12)	
N3	0.1204 (3)	0.20862 (12)	0.7578 (3)	0.0376 (8)	
N4	0.0040 (3)	0.30328 (13)	0.7065 (3)	0.0487 (9)	
O1	0.0806 (3)	0.11293 (12)	0.8766 (3)	0.0520 (8)	
O2	0.3405 (3)	0.15789 (11)	0.9454 (3)	0.0471 (7)	

### Atomic displacement parameters (Å^2^)

**Table:**

	*U*^11^	*U*^22^	*U*^33^	*U*^12^	*U*^13^	*U*^23^
Ir1	0.03609 (9)	0.03515 (9)	0.04216 (10)	0.00151 (8)	0.01377 (7)	0.00015 (8)
C1	0.073 (4)	0.070 (3)	0.056 (3)	0.004 (3)	0.003 (3)	0.005 (3)
C2	0.058 (3)	0.050 (3)	0.049 (3)	0.005 (2)	0.014 (2)	−0.001 (2)
C3	0.075 (4)	0.092 (4)	0.045 (3)	−0.015 (3)	0.022 (3)	0.002 (3)
C4	0.069 (3)	0.057 (3)	0.059 (3)	−0.005 (3)	0.035 (3)	−0.004 (2)
C5	0.085 (4)	0.094 (4)	0.080 (4)	−0.026 (3)	0.051 (3)	−0.010 (3)
C6	0.067 (3)	0.048 (3)	0.076 (4)	0.009 (2)	0.044 (3)	0.013 (2)
C7	0.090 (4)	0.043 (3)	0.105 (5)	0.017 (3)	0.062 (4)	0.023 (3)
C8	0.054 (3)	0.041 (2)	0.052 (3)	0.005 (2)	0.023 (2)	0.000 (2)
C9	0.040 (2)	0.038 (2)	0.041 (2)	0.0029 (18)	0.0147 (19)	−0.0014 (18)
C10	0.166 (7)	0.056 (4)	0.203 (8)	0.029 (4)	0.145 (7)	0.050 (4)
C11	0.046 (3)	0.041 (2)	0.055 (3)	0.007 (2)	0.018 (2)	0.007 (2)
C12	0.062 (3)	0.064 (3)	0.062 (3)	0.007 (3)	0.016 (3)	−0.005 (3)
C13	0.050 (3)	0.087 (4)	0.086 (4)	−0.004 (3)	0.016 (3)	−0.005 (3)
C14	0.062 (4)	0.078 (4)	0.103 (5)	0.016 (3)	0.044 (3)	0.004 (3)
C15	0.078 (4)	0.065 (3)	0.081 (4)	0.011 (3)	0.043 (3)	−0.009 (3)
C16	0.060 (3)	0.046 (3)	0.071 (3)	0.006 (2)	0.027 (3)	−0.001 (2)
C17	0.037 (2)	0.040 (2)	0.039 (2)	−0.0041 (18)	0.0057 (19)	−0.0006 (18)
C18	0.047 (2)	0.045 (2)	0.044 (3)	−0.004 (2)	0.019 (2)	−0.002 (2)
C19	0.053 (3)	0.054 (3)	0.047 (3)	−0.007 (2)	0.023 (2)	0.002 (2)
C20	0.049 (3)	0.047 (3)	0.048 (3)	−0.008 (2)	0.009 (2)	0.010 (2)
C21	0.043 (2)	0.041 (2)	0.052 (3)	0.004 (2)	0.009 (2)	0.007 (2)
C22	0.037 (2)	0.040 (2)	0.034 (2)	−0.0005 (18)	0.0063 (18)	−0.0029 (17)
C23	0.043 (2)	0.045 (2)	0.039 (2)	0.002 (2)	0.009 (2)	−0.0051 (19)
C24	0.050 (3)	0.043 (2)	0.043 (3)	0.003 (2)	0.015 (2)	−0.0064 (19)
C25	0.040 (2)	0.045 (2)	0.045 (3)	0.002 (2)	0.010 (2)	−0.003 (2)
C26	0.035 (2)	0.043 (2)	0.042 (2)	−0.0021 (18)	0.0081 (19)	−0.0049 (19)
C27	0.083 (4)	0.043 (3)	0.060 (3)	0.010 (3)	0.007 (3)	−0.009 (2)
C28	0.038 (2)	0.045 (2)	0.048 (3)	0.0048 (19)	0.009 (2)	0.000 (2)
C29	0.046 (3)	0.096 (4)	0.066 (4)	0.012 (3)	0.017 (3)	0.024 (3)
C30	0.038 (3)	0.108 (5)	0.080 (4)	0.014 (3)	0.012 (3)	0.027 (4)
C31	0.050 (3)	0.079 (4)	0.060 (3)	0.012 (3)	−0.003 (3)	0.012 (3)
C32	0.063 (3)	0.115 (5)	0.048 (3)	0.010 (3)	0.010 (3)	0.006 (3)
C33	0.048 (3)	0.106 (4)	0.059 (3)	0.016 (3)	0.019 (3)	0.002 (3)
C34	0.039 (2)	0.043 (2)	0.049 (3)	−0.003 (2)	0.007 (2)	−0.006 (2)
C35	0.058 (3)	0.049 (3)	0.077 (4)	0.006 (2)	−0.003 (3)	−0.017 (3)
C36	0.065 (3)	0.064 (3)	0.076 (4)	−0.013 (3)	−0.012 (3)	−0.018 (3)
C37	0.072 (3)	0.044 (3)	0.068 (3)	−0.011 (3)	0.013 (3)	−0.014 (2)
C38	0.054 (3)	0.037 (2)	0.058 (3)	0.001 (2)	0.017 (2)	−0.006 (2)
C39	0.037 (2)	0.042 (2)	0.047 (3)	−0.0039 (18)	0.016 (2)	0.0003 (19)
N1	0.048 (2)	0.0341 (18)	0.046 (2)	0.0037 (15)	0.0191 (17)	0.0035 (15)
N2	0.081 (3)	0.039 (2)	0.093 (3)	0.016 (2)	0.054 (3)	0.016 (2)
N3	0.0367 (18)	0.0359 (18)	0.042 (2)	0.0017 (15)	0.0136 (16)	−0.0035 (15)
N4	0.050 (2)	0.044 (2)	0.051 (2)	0.0083 (17)	0.0122 (19)	−0.0024 (18)
O1	0.0475 (18)	0.0573 (19)	0.057 (2)	−0.0063 (15)	0.0237 (16)	−0.0032 (16)
O2	0.0431 (17)	0.0506 (17)	0.0455 (18)	−0.0010 (14)	0.0086 (14)	0.0029 (14)

### Geometric parameters (Å, º)

**Table:**

Ir1—N1	2.035 (3)	C17—C22	1.433 (6)
Ir1—N3	2.035 (3)	C18—C19	1.394 (6)
Ir1—O1	2.162 (3)	C18—H8A	0.9300
Ir1—O2	2.173 (3)	C19—C20	1.381 (6)
Ir1—C22	2.006 (4)	C19—H4A	0.9300
Ir1—C39	1.999 (4)	C20—C21	1.386 (6)
C1—C2	1.508 (7)	C20—H10A	0.9300
C1—H22A	0.9600	C21—C22	1.401 (6)
C1—H22B	0.9600	C21—H11A	0.9300
C1—H22C	0.9600	C23—N3	1.338 (5)
C2—O2	1.285 (5)	C23—C24	1.390 (6)
C2—C3	1.401 (7)	C23—H28A	0.9300
C3—C4	1.375 (7)	C24—N4	1.340 (5)
C3—H20A	0.9300	C24—C27	1.503 (6)
C4—O1	1.274 (6)	C25—N4	1.344 (5)
C4—C5	1.525 (6)	C25—C26	1.409 (6)
C5—H19A	0.9600	C25—C28	1.495 (6)
C5—H19B	0.9600	C26—N3	1.373 (5)
C5—H19C	0.9600	C26—C34	1.479 (6)
C6—N1	1.349 (5)	C27—H34A	0.9600
C6—C7	1.374 (6)	C27—H34B	0.9600
C6—H2A	0.9300	C27—H34C	0.9600
C7—N2	1.346 (6)	C28—C33	1.367 (6)
C7—C10	1.509 (7)	C28—C29	1.385 (6)
C8—N2	1.347 (5)	C29—C30	1.385 (7)
C8—C9	1.413 (6)	C29—H33A	0.9300
C8—C11	1.495 (6)	C30—C31	1.358 (7)
C9—N1	1.373 (5)	C30—H35A	0.9300
C9—C17	1.471 (6)	C31—C32	1.356 (7)
C10—H39A	0.9600	C31—H36A	0.9300
C10—H39B	0.9600	C32—C33	1.388 (7)
C10—H39C	0.9600	C32—H37A	0.9300
C11—C16	1.385 (6)	C33—H38A	0.9300
C11—C12	1.391 (6)	C34—C35	1.402 (6)
C12—C13	1.395 (6)	C34—C39	1.419 (6)
C12—H17A	0.9300	C35—C36	1.387 (7)
C13—C14	1.373 (8)	C35—H25A	0.9300
C13—H13A	0.9300	C36—C37	1.385 (7)
C14—C15	1.372 (8)	C36—H30A	0.9300
C14—H14A	0.9300	C37—C38	1.371 (6)
C15—C16	1.380 (6)	C37—H24A	0.9300
C15—H15A	0.9300	C38—C39	1.404 (6)
C16—H16A	0.9300	C38—H23A	0.9300
C17—C18	1.407 (5)		
			
C39—Ir1—C22	90.85 (16)	C17—C18—H8A	120.1
C39—Ir1—N3	80.27 (15)	C20—C19—C18	119.9 (4)
C22—Ir1—N3	95.01 (15)	C20—C19—H4A	120.1
C39—Ir1—N1	96.76 (15)	C18—C19—H4A	120.1
C22—Ir1—N1	80.28 (14)	C19—C20—C21	120.7 (4)
N3—Ir1—N1	174.44 (12)	C19—C20—H10A	119.6
C39—Ir1—O1	87.99 (14)	C21—C20—H10A	119.6
C22—Ir1—O1	174.49 (13)	C20—C21—C22	121.9 (4)
N3—Ir1—O1	90.10 (12)	C20—C21—H11A	119.1
N1—Ir1—O1	94.50 (12)	C22—C21—H11A	119.1
C39—Ir1—O2	174.37 (13)	C21—C22—C17	116.7 (4)
C22—Ir1—O2	93.77 (14)	C21—C22—Ir1	128.2 (3)
N3—Ir1—O2	96.12 (12)	C17—C22—Ir1	115.0 (3)
N1—Ir1—O2	87.19 (12)	N3—C23—C24	121.4 (4)
O1—Ir1—O2	87.71 (11)	N3—C23—H28A	119.3
C2—C1—H22A	109.5	C24—C23—H28A	119.3
C2—C1—H22B	109.5	N4—C24—C23	120.7 (4)
H22A—C1—H22B	109.5	N4—C24—C27	117.2 (4)
C2—C1—H22C	109.5	C23—C24—C27	122.0 (4)
H22A—C1—H22C	109.5	N4—C25—C26	122.2 (4)
H22B—C1—H22C	109.5	N4—C25—C28	113.2 (4)
O2—C2—C3	126.1 (5)	C26—C25—C28	124.6 (4)
O2—C2—C1	116.4 (4)	N3—C26—C25	118.0 (4)
C3—C2—C1	117.5 (4)	N3—C26—C34	112.8 (3)
C4—C3—C2	128.9 (5)	C25—C26—C34	129.1 (4)
C4—C3—H20A	115.5	C24—C27—H34A	109.5
C2—C3—H20A	115.5	C24—C27—H34B	109.5
O1—C4—C3	127.3 (4)	H34A—C27—H34B	109.5
O1—C4—C5	114.2 (5)	C24—C27—H34C	109.5
C3—C4—C5	118.5 (5)	H34A—C27—H34C	109.5
C4—C5—H19A	109.5	H34B—C27—H34C	109.5
C4—C5—H19B	109.5	C33—C28—C29	118.3 (4)
H19A—C5—H19B	109.5	C33—C28—C25	120.1 (4)
C4—C5—H19C	109.5	C29—C28—C25	121.4 (4)
H19A—C5—H19C	109.5	C28—C29—C30	120.3 (5)
H19B—C5—H19C	109.5	C28—C29—H33A	119.8
N1—C6—C7	121.7 (4)	C30—C29—H33A	119.8
N1—C6—H2A	119.1	C31—C30—C29	119.9 (5)
C7—C6—H2A	119.1	C31—C30—H35A	120.1
N2—C7—C6	120.5 (4)	C29—C30—H35A	120.1
N2—C7—C10	117.3 (4)	C32—C31—C30	120.9 (5)
C6—C7—C10	122.1 (4)	C32—C31—H36A	119.5
N2—C8—C9	122.5 (4)	C30—C31—H36A	119.5
N2—C8—C11	113.6 (4)	C31—C32—C33	119.2 (5)
C9—C8—C11	123.8 (4)	C31—C32—H37A	120.4
N1—C9—C8	117.1 (4)	C33—C32—H37A	120.4
N1—C9—C17	113.4 (3)	C28—C33—C32	121.3 (5)
C8—C9—C17	129.5 (4)	C28—C33—H38A	119.4
C7—C10—H39A	109.5	C32—C33—H38A	119.4
C7—C10—H39B	109.5	C35—C34—C39	120.7 (4)
H39A—C10—H39B	109.5	C35—C34—C26	125.0 (4)
C7—C10—H39C	109.5	C39—C34—C26	114.3 (4)
H39A—C10—H39C	109.5	C36—C35—C34	120.1 (5)
H39B—C10—H39C	109.5	C36—C35—H25A	120.0
C16—C11—C12	118.8 (4)	C34—C35—H25A	120.0
C16—C11—C8	119.7 (4)	C37—C36—C35	119.3 (5)
C12—C11—C8	121.5 (4)	C37—C36—H30A	120.3
C11—C12—C13	120.5 (5)	C35—C36—H30A	120.3
C11—C12—H17A	119.8	C38—C37—C36	121.2 (4)
C13—C12—H17A	119.8	C38—C37—H24A	119.4
C14—C13—C12	119.3 (5)	C36—C37—H24A	119.4
C14—C13—H13A	120.4	C37—C38—C39	121.6 (4)
C12—C13—H13A	120.4	C37—C38—H23A	119.2
C15—C14—C13	120.9 (5)	C39—C38—H23A	119.2
C15—C14—H14A	119.6	C38—C39—C34	117.0 (4)
C13—C14—H14A	119.6	C38—C39—Ir1	127.6 (3)
C14—C15—C16	119.9 (5)	C34—C39—Ir1	115.2 (3)
C14—C15—H15A	120.1	C6—N1—C9	119.5 (3)
C16—C15—H15A	120.1	C6—N1—Ir1	123.7 (3)
C15—C16—C11	120.7 (5)	C9—N1—Ir1	116.8 (3)
C15—C16—H16A	119.6	C7—N2—C8	118.3 (4)
C11—C16—H16A	119.6	C23—N3—C26	119.3 (3)
C18—C17—C22	120.7 (4)	C23—N3—Ir1	123.7 (3)
C18—C17—C9	125.2 (4)	C26—N3—Ir1	116.9 (3)
C22—C17—C9	114.0 (3)	C24—N4—C25	118.4 (4)
C19—C18—C17	119.7 (4)	C4—O1—Ir1	124.7 (3)
C19—C18—H8A	120.1	C2—O2—Ir1	124.2 (3)
			
O2—C2—C3—C4	0.6 (9)	C39—C34—C35—C36	−2.8 (8)
C1—C2—C3—C4	179.9 (5)	C26—C34—C35—C36	177.5 (5)
C2—C3—C4—O1	3.7 (10)	C34—C35—C36—C37	−0.6 (9)
C2—C3—C4—C5	−177.7 (5)	C35—C36—C37—C38	2.1 (9)
N1—C6—C7—N2	4.8 (9)	C36—C37—C38—C39	−0.3 (8)
N1—C6—C7—C10	−177.1 (6)	C37—C38—C39—C34	−2.9 (6)
N2—C8—C9—N1	6.4 (7)	C37—C38—C39—Ir1	172.9 (4)
C11—C8—C9—N1	−171.2 (4)	C35—C34—C39—C38	4.4 (6)
N2—C8—C9—C17	−173.0 (4)	C26—C34—C39—C38	−175.8 (4)
C11—C8—C9—C17	9.5 (7)	C35—C34—C39—Ir1	−171.9 (4)
N2—C8—C11—C16	58.3 (6)	C26—C34—C39—Ir1	7.8 (5)
C9—C8—C11—C16	−123.9 (5)	C22—Ir1—C39—C38	84.3 (4)
N2—C8—C11—C12	−119.4 (5)	N3—Ir1—C39—C38	179.2 (4)
C9—C8—C11—C12	58.3 (7)	N1—Ir1—C39—C38	4.0 (4)
C16—C11—C12—C13	0.5 (7)	O1—Ir1—C39—C38	−90.3 (4)
C8—C11—C12—C13	178.3 (5)	C22—Ir1—C39—C34	−99.8 (3)
C11—C12—C13—C14	−0.5 (8)	N3—Ir1—C39—C34	−4.9 (3)
C12—C13—C14—C15	1.5 (9)	N1—Ir1—C39—C34	179.9 (3)
C13—C14—C15—C16	−2.5 (9)	O1—Ir1—C39—C34	85.6 (3)
C14—C15—C16—C11	2.6 (8)	C7—C6—N1—C9	−1.3 (8)
C12—C11—C16—C15	−1.6 (7)	C7—C6—N1—Ir1	−179.5 (4)
C8—C11—C16—C15	−179.4 (5)	C8—C9—N1—C6	−4.0 (6)
N1—C9—C17—C18	−169.3 (4)	C17—C9—N1—C6	175.5 (4)
C8—C9—C17—C18	10.1 (7)	C8—C9—N1—Ir1	174.3 (3)
N1—C9—C17—C22	7.7 (5)	C17—C9—N1—Ir1	−6.3 (4)
C8—C9—C17—C22	−172.9 (4)	C39—Ir1—N1—C6	−89.6 (4)
C22—C17—C18—C19	4.9 (6)	C22—Ir1—N1—C6	−179.3 (4)
C9—C17—C18—C19	−178.3 (4)	O1—Ir1—N1—C6	−1.1 (4)
C17—C18—C19—C20	−0.5 (7)	O2—Ir1—N1—C6	86.4 (4)
C18—C19—C20—C21	−2.8 (7)	C39—Ir1—N1—C9	92.3 (3)
C19—C20—C21—C22	1.6 (7)	C22—Ir1—N1—C9	2.5 (3)
C20—C21—C22—C17	2.7 (6)	O1—Ir1—N1—C9	−179.2 (3)
C20—C21—C22—Ir1	−174.2 (3)	O2—Ir1—N1—C9	−91.8 (3)
C18—C17—C22—C21	−5.9 (6)	C6—C7—N2—C8	−2.4 (9)
C9—C17—C22—C21	177.0 (4)	C10—C7—N2—C8	179.3 (6)
C18—C17—C22—Ir1	171.4 (3)	C9—C8—N2—C7	−3.1 (8)
C9—C17—C22—Ir1	−5.7 (4)	C11—C8—N2—C7	174.6 (5)
C39—Ir1—C22—C21	82.1 (4)	C24—C23—N3—C26	0.5 (6)
N3—Ir1—C22—C21	1.8 (4)	C24—C23—N3—Ir1	175.7 (3)
N1—Ir1—C22—C21	178.8 (4)	C25—C26—N3—C23	1.4 (5)
O2—Ir1—C22—C21	−94.7 (4)	C34—C26—N3—C23	178.3 (4)
C39—Ir1—C22—C17	−94.8 (3)	C25—C26—N3—Ir1	−174.1 (3)
N3—Ir1—C22—C17	−175.1 (3)	C34—C26—N3—Ir1	2.8 (4)
N1—Ir1—C22—C17	1.9 (3)	C39—Ir1—N3—C23	−174.3 (3)
O2—Ir1—C22—C17	88.4 (3)	C22—Ir1—N3—C23	−84.3 (3)
N3—C23—C24—N4	−1.0 (6)	O1—Ir1—N3—C23	97.8 (3)
N3—C23—C24—C27	−178.1 (4)	O2—Ir1—N3—C23	10.1 (3)
N4—C25—C26—N3	−2.8 (6)	C39—Ir1—N3—C26	1.0 (3)
C28—C25—C26—N3	174.3 (4)	C22—Ir1—N3—C26	91.0 (3)
N4—C25—C26—C34	−179.2 (4)	O1—Ir1—N3—C26	−86.9 (3)
C28—C25—C26—C34	−2.1 (7)	O2—Ir1—N3—C26	−174.6 (3)
N4—C25—C28—C33	96.8 (5)	C23—C24—N4—C25	−0.4 (6)
C26—C25—C28—C33	−80.6 (6)	C27—C24—N4—C25	176.8 (4)
N4—C25—C28—C29	−77.3 (6)	C26—C25—N4—C24	2.3 (6)
C26—C25—C28—C29	105.4 (5)	C28—C25—N4—C24	−175.2 (4)
C33—C28—C29—C30	2.3 (8)	C3—C4—O1—Ir1	2.3 (7)
C25—C28—C29—C30	176.5 (5)	C5—C4—O1—Ir1	−176.4 (3)
C28—C29—C30—C31	−0.6 (9)	C39—Ir1—O1—C4	176.2 (4)
C29—C30—C31—C32	−2.4 (10)	N3—Ir1—O1—C4	−103.6 (4)
C30—C31—C32—C33	3.4 (9)	N1—Ir1—O1—C4	79.6 (4)
C29—C28—C33—C32	−1.3 (8)	O2—Ir1—O1—C4	−7.4 (4)
C25—C28—C33—C32	−175.5 (5)	C3—C2—O2—Ir1	−9.8 (7)
C31—C32—C33—C28	−1.6 (9)	C1—C2—O2—Ir1	171.0 (3)
N3—C26—C34—C35	172.9 (4)	C22—Ir1—O2—C2	−163.8 (3)
C25—C26—C34—C35	−10.5 (7)	N3—Ir1—O2—C2	100.8 (3)
N3—C26—C34—C39	−6.8 (5)	N1—Ir1—O2—C2	−83.7 (3)
C25—C26—C34—C39	169.7 (4)	O1—Ir1—O2—C2	10.9 (3)
